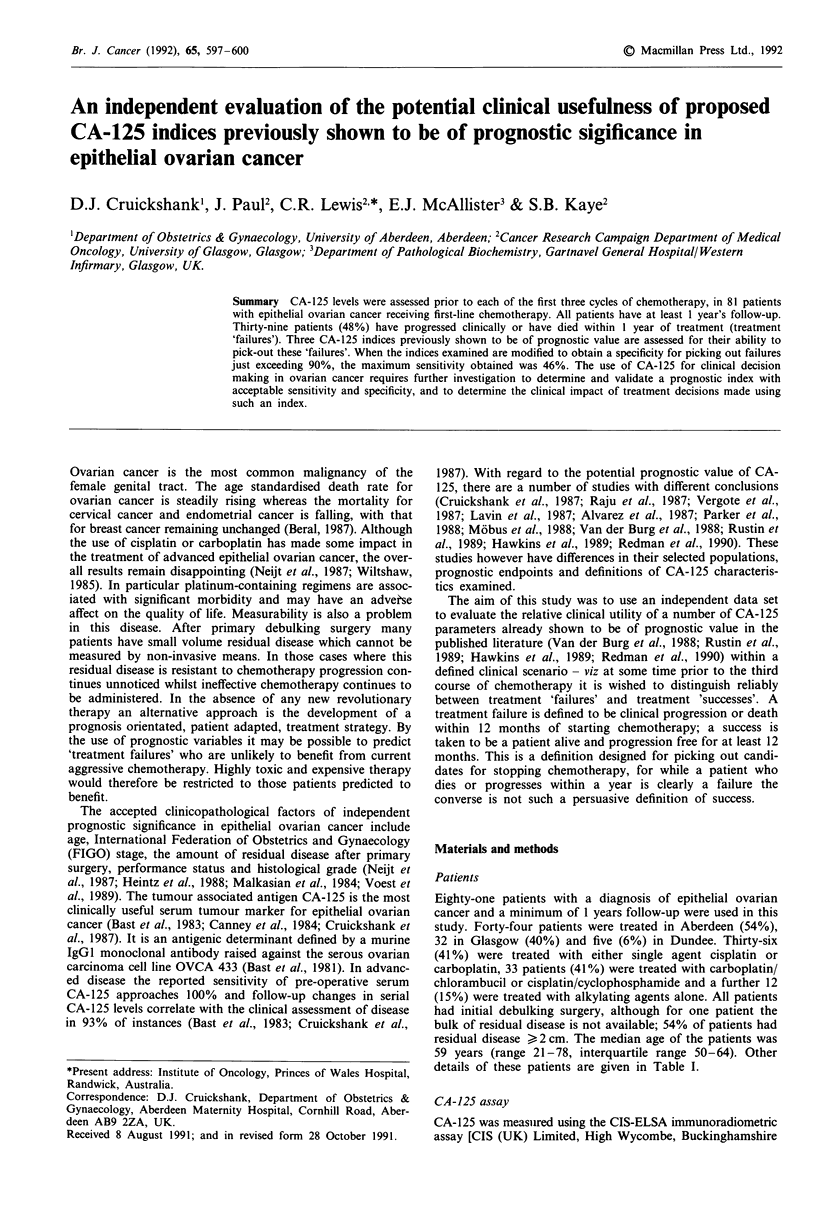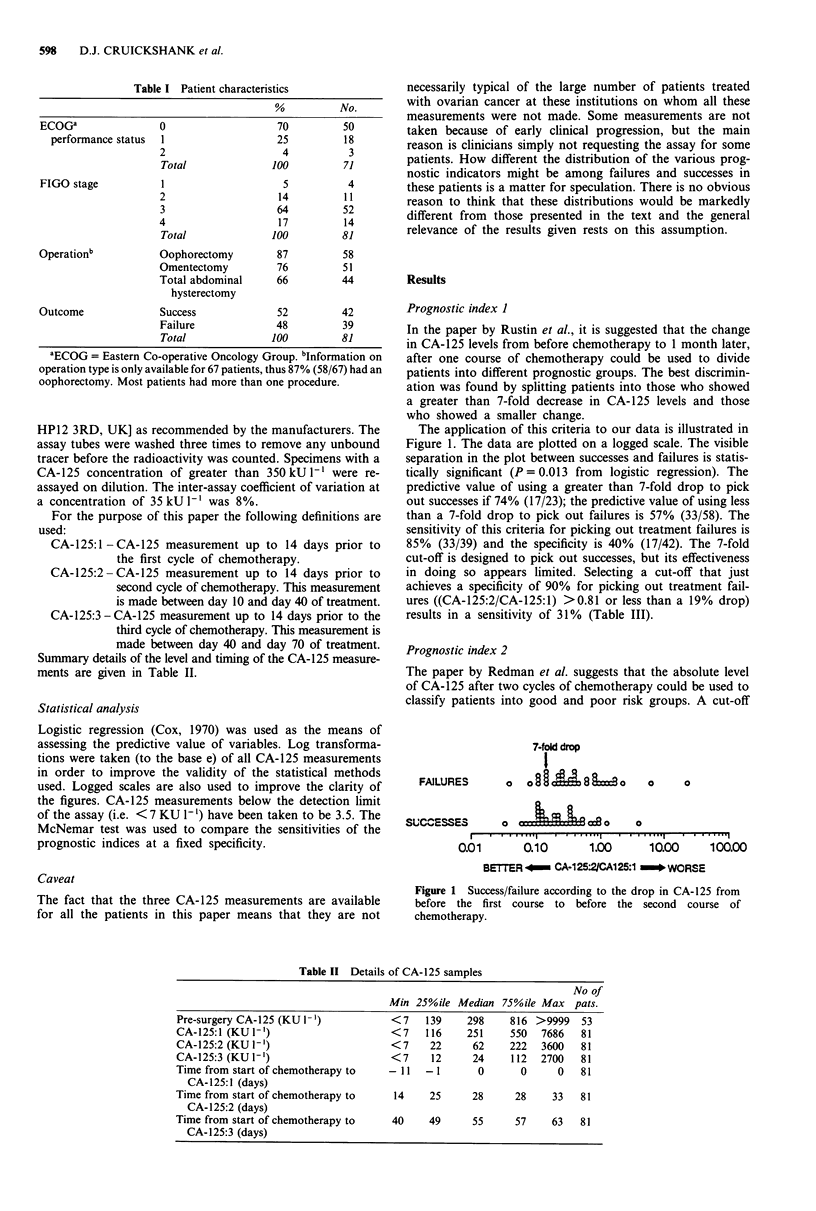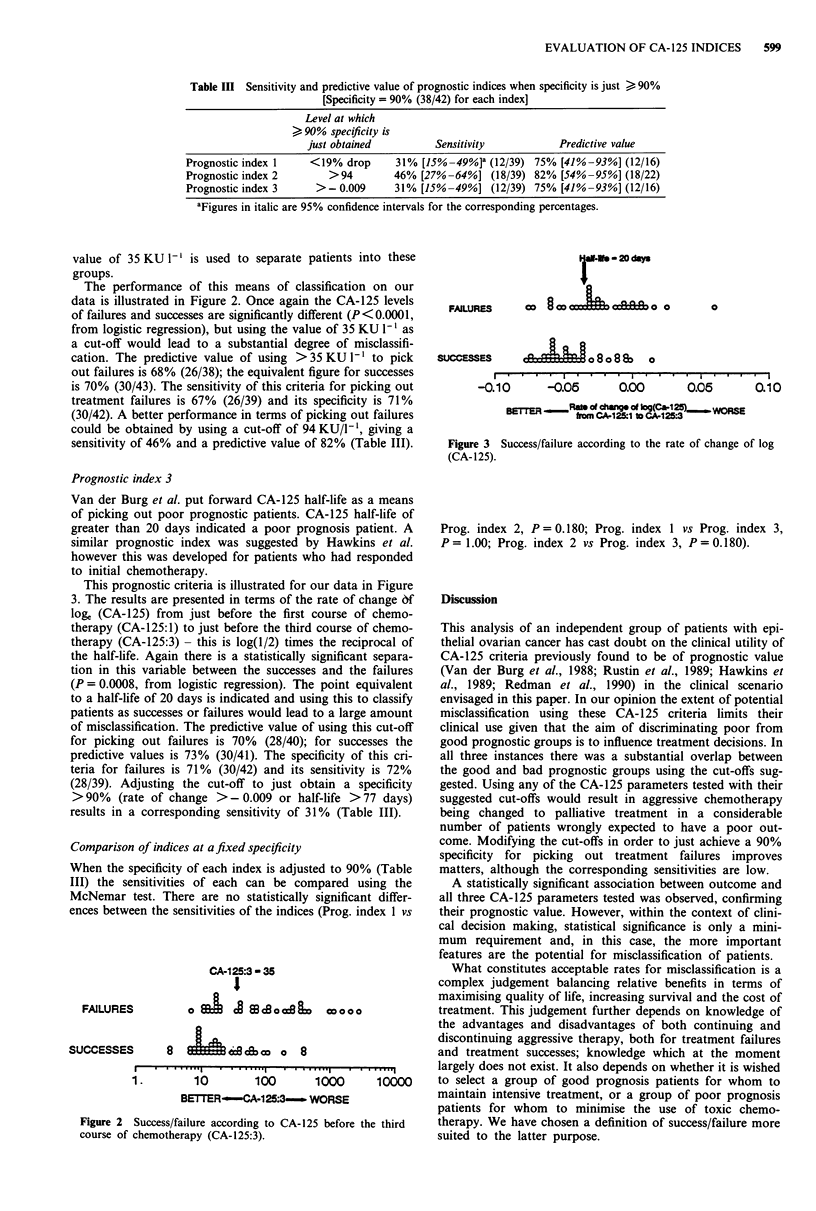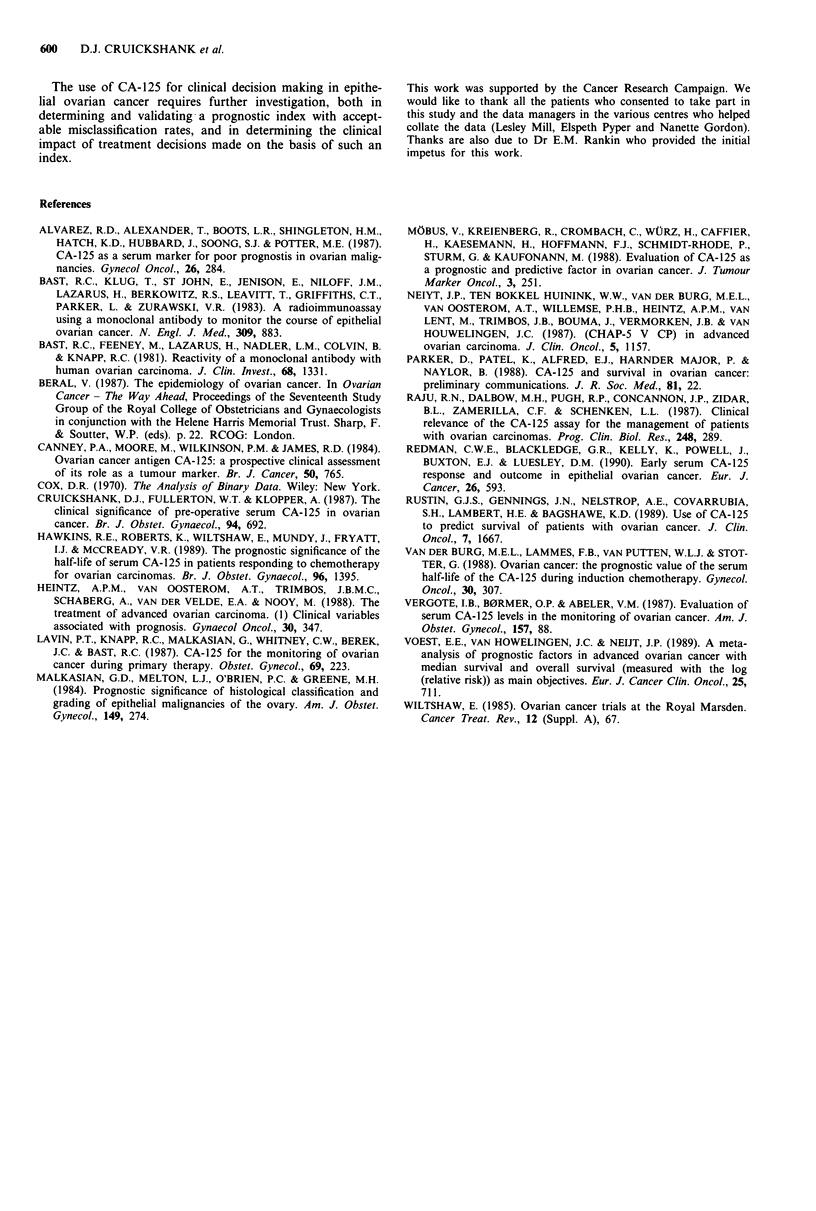# An independent evaluation of the potential clinical usefulness of proposed CA-125 indices previously shown to be of prognostic significance in epithelial ovarian cancer.

**DOI:** 10.1038/bjc.1992.121

**Published:** 1992-04

**Authors:** D. J. Cruickshank, J. Paul, C. R. Lewis, E. J. McAllister, S. B. Kaye

**Affiliations:** Department of Obstetrics & Gynaecology, University of Aberdeen, UK.

## Abstract

CA-125 levels were assessed prior to each of the first three cycles of chemotherapy, in 81 patients with epithelial ovarian cancer receiving first-line chemotherapy. All patients have at least 1 year's follow-up. Thirty-nine patients (48%) have progressed clinically or have died within 1 year of treatment (treatment 'failures'). Three CA-125 indices previously shown to be of prognostic value are assessed for their ability to pick-out these 'failures'. When the indices examined are modified to obtain a specificity for picking out failures just exceeding 90%, the maximum sensitivity obtained was 46%. The use of CA-125 for clinical decision making in ovarian cancer requires further investigation to determine and validate a prognostic index with acceptable sensitivity and specificity, and to determine the clinical impact of treatment decisions made using such an index.


Br. J. Cancer (1992), 65, 597-600 © Macmillan Press Ltd., 1992

### An independent evaluation of the potential clinical usefulness of proposed CA-125 indices previously shown to be of prognostic sigificance in epithelial ovarian cancer

D.J. Cruickshank[1], J. Paul[2], C.R. Lewis[2,*], E.J. McAllister[3] & S.B. Kaye[2]

[1]*Department of Obstetrics & Gynaecology, University of Aberdeen, Aberdeen;* [2]*Cancer Research Campaign Department of Medical Oncology, University of Glasgow, Glasgow;* [3]*Department of Pathological Biochemistry, Gartnavel General Hospital/Western Infirmary, Glasgow, UK.*

**Summary** CA-125 levels were assessed prior to each of the first three cycles of chemotherapy, in 81 patients with epithelial ovarian cancer receiving first-line chemotherapy. All patients have at least 1 year's follow-up. Thirty-nine patients (48%) have progressed clinically or have died within 1 year of treatment (treatment 'failures'). Three CA-125 indices previously shown to be of prognostic value are assessed for their ability to pick-out these 'failures'. When the indices examined are modified to obtain a specificity for picking out failures just exceeding 90%, the maximum sensitivity obtained was 46%. The use of CA-125 for clinical decision making in ovarian cancer requires further investigation to determine and validate a prognostic index with acceptable sensitivity and specificity, and to determine the clinical impact of treatment decisions made using such an index.

Ovarian cancer is the most common malignancy of the female genital tract. The age standardised death rate for ovarian cancer is steadily rising whereas the mortality for cervical cancer and endometrial cancer is falling, with that for breast cancer remaining unchanged (Beral, 1987). Although the use of cisplatin or carboplatin has made some impact in the treatment of advanced epithelial ovarian cancer, the overall results remain disappointing (Neijt *et al.*, 1987; Wiltshaw, 1985). In particular platinum-containing regimens are associated with significant morbidity and may have an adverse affect on the quality of life. Measurability is also a problem in this disease. After primary debulking surgery many patients have small volume residual disease which cannot be measured by non-invasive means. In those cases where this residual disease is resistant to chemotherapy progression continues unnoticed whilst ineffective chemotherapy continues to be administered. In the absence of any new revolutionary therapy an alternative approach is the development of a prognosis orientated, patient adapted, treatment strategy. By the use of prognostic variables it may be possible to predict 'treatment failures' who are unlikely to benefit from current aggressive chemotherapy. Highly toxic and expensive therapy would therefore be restricted to those patients predicted to benefit.

The accepted clinicopathological factors of independent prognostic significance in epithelial ovarian cancer include age, International Federation of Obstetrics and Gynaecology (FIGO) stage, the amount of residual disease after primary surgery, performance status and histological grade (Neijt *et al.*, 1987; Heintz *et al.*, 1988; Malkasian *et al.*, 1984; Voest *et al.*, 1989). The tumour associated antigen CA-125 is the most clinically useful serum tumour marker for epithelial ovarian cancer (Bast *et al.*, 1983; Canney *et al.*, 1984; Cruickshank *et al.*, 1987). It is an antigenic determinant defined by a murine IgG1 monoclonal antibody raised against the serous ovarian carcinoma cell line OVCA 433 (Bast *et al.*, 1981). In advanced disease the reported sensitivity of pre-operative serum CA-125 approaches 100% and follow-up changes in serial CA-125 levels correlate with the clinical assessment of disease in 93% of instances (Bast *et al.*, 1983; Cruickshank *et al.*, 1987). With regard to the potential prognostic value of CA-125, there are a number of studies with different conclusions (Cruickshank *et al.*, 1987; Raju *et al.*, 1987; Vergote *et al.*, 1987; Lavin *et al.*, 1987; Alvarez *et al.*, 1987; Parker *et al.*, 1988; Möbus *et al.*, 1988; Van der Burg *et al.*, 1988; Rustin *et al.*, 1989; Hawkins *et al.*, 1989; Redman *et al.*, 1990). These studies however have differences in their selected populations, prognostic endpoints and definitions of CA-125 characteristics examined.

The aim of this study was to use an independent data set to evaluate the relative clinical utility of a number of CA-125 parameters already shown to be of prognostic value in the published literature (Van der Burg *et al.*, 1988; Rustin *et al.*, 1989; Hawkins *et al.*, 1989; Redman *et al.*, 1990) within a defined clinical scenario – *viz* at some time prior to the third course of chemotherapy it is wished to distinguish reliably between treatment 'failures' and treatment 'successes'. A treatment failure is defined to be clinical progression or death within 12 months of starting chemotherapy; a success is taken to be a patient alive and progression free for at least 12 months. This is a definition designed for picking out candidates for stopping chemotherapy, for while a patient who dies or progresses within a year is clearly a failure the converse is not such a persuasive definition of success.

### Materials and methods

### *Patients*

Eighty-one patients with a diagnosis of epithelial ovarian cancer and a minimum of 1 years follow-up were used in this study. Forty-four patients were treated in Aberdeen (54%), 32 in Glasgow (40%) and five (6%) in Dundee. Thirty-six (41%) were treated with either single agent cisplatin or carboplatin, 33 patients (41%) were treated with carboplatin/chlorambucil or cisplatin/cyclophosphamide and a further 12 (15%) were treated with alkylating agents alone. All patients had initial debulking surgery, although for one patient the bulk of residual disease is not available; 54% of patients had residual disease ⩾2 cm. The median age of the patients was 59 years (range 21–78, interquartile range 50–64). Other details of these patients are given in Table I.

### *CA-125 assay*

CA-125 was measured using the CIS-ELSA immunoradiometric assay [CIS (UK) Limited, High Wycombe, Buckinghamshire

*Present address: Institute of Oncology, Princes of Wales Hospital, Randwick, Australia.
Correspondence: D.J. Cruickshank, Department of Obstetrics & Gynaecology, Aberdeen Maternity Hospital, Cornhill Road, Aberdeen AB9 2ZA, UK.
Received 8 August 1991; and in revised form 28 October 1991.

**Table I** Patient characteristics

| | | % | No. |
|---|---|---|---|
| ECOG[a] performance status | 0 | 70 | 50 |
| | 1 | 25 | 18 |
| | 2 | 4 | 3 |
| | *Total* | *100* | *71* |
| FIGO stage | 1 | 5 | 4 |
| | 2 | 14 | 11 |
| | 3 | 64 | 52 |
| | 4 | 17 | 14 |
| | *Total* | *100* | *81* |
| Operation[b] | Oophorectomy | 87 | 58 |
| | Omentectomy | 76 | 51 |
| | Total abdominal hysterectomy | 66 | 44 |
| Outcome | Success | 52 | 42 |
| | Failure | 48 | 39 |
| | *Total* | *100* | *81* |

[a]ECOG = Eastern Co-operative Oncology Group. [b]Information on operation type is only available for 67 patients, thus 87% (58/67) had an oophorectomy. Most patients had more than one procedure.

HP12 3RD, UK] as recommended by the manufacturers. The assay tubes were washed three times to remove any unbound tracer before the radioactivity was counted. Specimens with a CA-125 concentration of greater than 350 $kU\,l^{-1}$ were re-assayed on dilution. The inter-assay coefficient of variation at a concentration of 35 $kU\,l^{-1}$ was 8%.

For the purpose of this paper the following definitions are used:

CA-125:1 – CA-125 measurement up to 14 days prior to the first cycle of chemotherapy.

CA-125:2 – CA-125 measurement up to 14 days prior to second cycle of chemotherapy. This measurement is made between day 10 and day 40 of treatment.

CA-125:3 – CA-125 measurement up to 14 days prior to the third cycle of chemotherapy. This measurement is made between day 40 and day 70 of treatment.

Summary details of the level and timing of the CA-125 measurements are given in Table II.

### *Statistical analysis*

Logistic regression (Cox, 1970) was used as the means of assessing the predictive value of variables. Log transformations were taken (to the base e) of all CA-125 measurements in order to improve the validity of the statistical methods used. Logged scales are also used to improve the clarity of the figures. CA-125 measurements below the detection limit of the assay (i.e. $<7$ $KU\,l^{-1}$) have been taken to be 3.5. The McNemar test was used to compare the sensitivities of the prognostic indices at a fixed specificity.

### *Caveat*

The fact that the three CA-125 measurements are available for all the patients in this paper means that they are not necessarily typical of the large number of patients treated with ovarian cancer at these institutions on whom all these measurements were not made. Some measurements are not taken because of early clinical progression, but the main reason is clinicians simply not requesting the assay for some patients. How different the distribution of the various prognostic indicators might be among failures and successes in these patients is a matter for speculation. There is no obvious reason to think that these distributions would be markedly different from those presented in the text and the general relevance of the results given rests on this assumption.

### Results

### *Prognostic index 1*

In the paper by Rustin *et al.*, it is suggested that the change in CA-125 levels from before chemotherapy to 1 month later, after one course of chemotherapy could be used to divide patients into different prognostic groups. The best discrimination was found by splitting patients into those who showed a greater than 7-fold decrease in CA-125 levels and those who showed a smaller change.

The application of this criteria to our data is illustrated in Figure 1. The data are plotted on a logged scale. The visible separation in the plot between successes and failures is statistically significant ($P = 0.013$ from logistic regression). The predictive value of using a greater than 7-fold drop to pick out successes if 74% (17/23); the predictive value of using less than a 7-fold drop to pick out failures is 57% (33/58). The sensitivity of this criteria for picking out treatment failures is 85% (33/39) and the specificity is 40% (17/42). The 7-fold cut-off is designed to pick out successes, but its effectiveness in doing so appears limited. Selecting a cut-off that just achieves a specificity of 90% for picking out treatment failures ((CA-125:2/CA-125:1) $>0.81$ or less than a 19% drop) results in a sensitivity of 31% (Table III).

### *Prognostic index 2*

The paper by Redman *et al.* suggests that the absolute level of CA-125 after two cycles of chemotherapy could be used to classify patients into good and poor risk groups. A cut-off

**Figure 1** Success/failure according to the drop in CA-125 from before the first course to before the second course of chemotherapy.

**Table II** Details of CA-125 samples

| | Min | 25%ile | Median | 75%ile | Max | No of pats. |
|---|---|---|---|---|---|---|
| Pre-surgery CA-125 ($KU\,l^{-1}$) | $<7$ | 139 | 298 | 816 | $>9999$ | 53 |
| CA-125:1 ($KU\,l^{-1}$) | $<7$ | 116 | 251 | 550 | 7686 | 81 |
| CA-125:2 ($KU\,l^{-1}$) | $<7$ | 22 | 62 | 222 | 3600 | 81 |
| CA-125:3 ($KU\,l^{-1}$) | $<7$ | 12 | 24 | 112 | 2700 | 81 |
| Time from start of chemotherapy to CA-125:1 (days) | – 11 | – 1 | 0 | 0 | 0 | 81 |
| Time from start of chemotherapy to CA-125:2 (days) | 14 | 25 | 28 | 28 | 33 | 81 |
| Time from start of chemotherapy to CA-125:3 (days) | 40 | 49 | 55 | 57 | 63 | 81 |

**Table III** Sensitivity and predictive value of prognostic indices when specificity is just ⩾90% [Specificity = 90% (38/42) for each index]

| | *Level at which ⩾90% specificity is just obtained* | *Sensitivity* | *Predictive value* |
|---|---|---|---|
| Prognostic index 1 | <19% drop | 31% [*15%–49%*][a] (12/39) | 75% [*41%–93%*] (12/16) |
| Prognostic index 2 | >94 | 46% [*27%–64%*] (18/39) | 82% [*54%–95%*] (18/22) |
| Prognostic index 3 | > − 0.009 | 31% [*15%–49%*] (12/39) | 75% [*41%–93%*] (12/16) |

[a]Figures in italic are 95% confidence intervals for the corresponding percentages.

value of 35 KU $l^{-1}$ is used to separate patients into these groups.

The performance of this means of classification on our data is illustrated in Figure 2. Once again the CA-125 levels of failures and successes are significantly different ($P<0.0001$, from logistic regression), but using the value of 35 KU $l^{-1}$ as a cut-off would lead to a substantial degree of misclassification. The predictive value of using >35 KU $l^{-1}$ to pick out failures is 68% (26/38); the equivalent figure for successes is 70% (30/43). The sensitivity of this criteria for picking out treatment failures is 67% (26/39) and its specificity is 71% (30/42). A better performance in terms of picking out failures could be obtained by using a cut-off of 94 KU/$l^{-1}$, giving a sensitivity of 46% and a predictive value of 82% (Table III).

*Prognostic index 3*

Van der Burg *et al.* put forward CA-125 half-life as a means of picking out poor prognostic patients. CA-125 half-life of greater than 20 days indicated a poor prognosis patient. A similar prognostic index was suggested by Hawkins *et al.* however this was developed for patients who had responded to initial chemotherapy.

This prognostic criteria is illustrated for our data in Figure 3. The results are presented in terms of the rate of change of $\log_e$ (CA-125) from just before the first course of chemotherapy (CA-125:1) to just before the third course of chemotherapy (CA-125:3) – this is log(1/2) times the reciprocal of the half-life. Again there is a statistically significant separation in this variable between the successes and the failures ($P=0.0008$, from logistic regression). The point equivalent to a half-life of 20 days is indicated and using this to classify patients as successes or failures would lead to a large amount of misclassification. The predictive value of using this cut-off for picking out failures is 70% (28/40); for successes the predictive values is 73% (30/41). The specificity of this criteria for failures is 71% (30/42) and its sensitivity is 72% (28/39). Adjusting the cut-off to just obtain a specificity >90% (rate of change > − 0.009 or half-life >77 days) results in a corresponding sensitivity of 31% (Table III).

*Comparison of indices at a fixed specificity*

When the specificity of each index is adjusted to 90% (Table III) the sensitivities of each can be compared using the McNemar test. There are no statistically significant differences between the sensitivities of the indices (Prog. index 1 *vs* Prog. index 2, $P=0.180$; Prog. index 1 *vs* Prog. index 3, $P=1.00$; Prog. index 2 *vs* Prog. index 3, $P=0.180$).

**Figure 2** Success/failure according to CA-125 before the third course of chemotherapy (CA-125:3).

**Figure 3** Success/failure according to the rate of change of log (CA-125).

### Discussion

This analysis of an independent group of patients with epithelial ovarian cancer has cast doubt on the clinical utility of CA-125 criteria previously found to be of prognostic value (Van der Burg *et al.*, 1988; Rustin *et al.*, 1989; Hawkins *et al.*, 1989; Redman *et al.*, 1990) in the clinical scenario envisaged in this paper. In our opinion the extent of potential misclassification using these CA-125 criteria limits their clinical use given that the aim of discriminating poor from good prognostic groups is to influence treatment decisions. In all three instances there was a substantial overlap between the good and bad prognostic groups using the cut-offs suggested. Using any of the CA-125 parameters tested with their suggested cut-offs would result in aggressive chemotherapy being changed to palliative treatment in a considerable number of patients wrongly expected to have a poor outcome. Modifying the cut-offs in order to just achieve a 90% specificity for picking out treatment failures improves matters, although the corresponding sensitivities are low.

A statistically significant association between outcome and all three CA-125 parameters tested was observed, confirming their prognostic value. However, within the context of clinical decision making, statistical significance is only a minimum requirement and, in this case, the more important features are the potential for misclassification of patients.

What constitutes acceptable rates for misclassification is a complex judgement balancing relative benefits in terms of maximising quality of life, increasing survival and the cost of treatment. This judgement further depends on knowledge of the advantages and disadvantages of both continuing and discontinuing aggressive therapy, both for treatment failures and treatment successes; knowledge which at the moment largely does not exist. It also depends on whether it is wished to select a group of good prognosis patients for whom to maintain intensive treatment, or a group of poor prognosis patients for whom to minimise the use of toxic chemotherapy. We have chosen a definition of success/failure more suited to the latter purpose.

The use of CA-125 for clinical decision making in epithelial ovarian cancer requires further investigation, both in determining and validating a prognostic index with acceptable misclassification rates, and in determining the clinical impact of treatment decisions made on the basis of such an index.

This work was supported by the Cancer Research Campaign. We would like to thank all the patients who consented to take part in this study and the data managers in the various centres who helped collate the data (Lesley Mill, Elspeth Pyper and Nanette Gordon). Thanks are also due to Dr E.M. Rankin who provided the initial impetus for this work.

### References

ALVAREZ, R.D., ALEXANDER, T., BOOTS, L.R., SHINGLETON, H.M., HATCH, K.D., HUBBARD, J., SOONG, S.J. & POTTER, M.E. (1987). CA-125 as a serum marker for poor prognostis in ovarian malignancies. *Gynecol Oncol.*, **26**, 284.

BAST, R.C., KLUG, T., ST JOHN, E., JENISON, E., NILOFF, J.M., LAZARUS, H., BERKOWITZ, R.S., LEAVITT, T., GRIFFITHS, C.T., PARKER, L. & ZURAWSKI, V.R. (1983). A radioimmunoassay using a monoclonal antibody to monitor the course of epithelial ovarian cancer. *N. Engl. J. Med.*, **309**, 883.

BAST, R.C., FEENEY, M., LAZARUS, H., NADLER, L.M., COLVIN, B. & KNAPP, R.C. (1981). Reactivity of a monoclonal antibody with human ovarian carcinoma. *J. Clin. Invest.*, **68**, 1331.

BERAL, V. (1987). The epidemiology of ovarian cancer. In *Ovarian Cancer – The Way Ahead*, Proceedings of the Seventeenth Study Group of the Royal College of Obstetricians and Gynaecologists in conjunction with the Helene Harris Memorial Trust. Sharp, F. & Soutter, W.P. (eds). p. 22. RCOG: London.

CANNEY, P.A., MOORE, M., WILKINSON, P.M. & JAMES, R.D. (1984). Ovarian cancer antigen CA-125: a prospective clinical assessment of its role as a tumour marker. *Br. J. Cancer*, **50**, 765.

COX, D.R. (1970). *The Analysis of Binary Data*. Wiley: New York.

CRUICKSHANK, D.J., FULLERTON, W.T. & KLOPPER, A. (1987). The clinical significance of pre-operative serum CA-125 in ovarian cancer. *Br. J. Obstet. Gynaecol.*, **94**, 692.

HAWKINS, R.E., ROBERTS, K., WILTSHAW, E., MUNDY, J., FRYATT, I.J. & MCCREADY, V.R. (1989). The prognostic significance of the half-life of serum CA-125 in patients responding to chemotherapy for ovarian carcinomas. *Br. J. Obstet. Gynaecol.*, **96**, 1395.

HEINTZ, A.P.M., VAN OOSTEROM, A.T., TRIMBOS, J.B.M.C., SCHABERG, A., VAN DER VELDE, E.A. & NOOY, M. (1988). The treatment of advanced ovarian carcinoma. (1) Clinical variables associated with prognosis. *Gynaecol Oncol.*, **30**, 347.

LAVIN, P.T., KNAPP, R.C., MALKASIAN, G., WHITNEY, C.W., BEREK, J.C. & BAST, R.C. (1987). CA-125 for the monitoring of ovarian cancer during primary therapy. *Obstet. Gynecol.*, **69**, 223.

MALKASIAN, G.D., MELTON, L.J., O'BRIEN, P.C. & GREENE, M.H. (1984). Prognostic significance of histological classification and grading of epithelial malignancies of the ovary. *Am. J. Obstet. Gynecol.*, **149**, 274.

MÖBUS, V., KREIENBERG, R., CROMBACH, C., WÜRZ, H., CAFFIER, H., KAESEMANN, H., HOFFMANN, F.J., SCHMIDT-RHODE, P., STURM, G. & KAUFONANN, M. (1988). Evaluation of CA-125 as a prognostic and predictive factor in ovarian cancer. *J. Tumour Marker Oncol.*, **3**, 251.

NEIYT, J.P., TEN BOKKEL HUININK, W.W., VAN DER BURG, M.E.L., VAN OOSTEROM, A.T., WILLEMSE, P.H.B., HEINTZ, A.P.M., VAN LENT, M., TRIMBOS, J.B., BOUMA, J., VERMORKEN, J.B. & VAN HOUWELINGEN, J.C. (1987). (CHAP-5 V CP) in advanced ovarian carcinoma. *J. Clin. Oncol.*, **5**, 1157.

PARKER, D., PATEL, K., ALFRED, E.J., HARNDER MAJOR, P. & NAYLOR, B. (1988). CA-125 and survival in ovarian cancer: preliminary communications. *J. R. Soc. Med.*, **81**, 22.

RAJU, R.N., DALBOW, M.H., PUGH, R.P., CONCANNON, J.P., ZIDAR, B.L., ZAMERILLA, C.F. & SCHENKEN, L.L. (1987). Clinical relevance of the CA-125 assay for the management of patients with ovarian carcinomas. *Prog. Clin. Biol. Res.*, **248**, 289.

REDMAN, C.W.E., BLACKLEDGE, G.R., KELLY, K., POWELL, J., BUXTON, E.J. & LUESLEY, D.M. (1990). Early serum CA-125 response and outcome in epithelial ovarian cancer. *Eur. J. Cancer*, **26**, 593.

RUSTIN, G.J.S., GENNINGS, J.N., NELSTROP, A.E., COVARRUBIA, S.H., LAMBERT, H.E. & BAGSHAWE, K.D. (1989). Use of CA-125 to predict survival of patients with ovarian cancer. *J. Clin. Oncol.*, **7**, 1667.

VAN DER BURG, M.E.L., LAMMES, F.B., VAN PUTTEN, W.L.J. & STOTTER, G. (1988). Ovarian cancer: the prognostic value of the serum half-life of the CA-125 during induction chemotherapy. *Gynecol. Oncol.*, **30**, 307.

VERGOTE, I.B., BØRMER, O.P. & ABELER, V.M. (1987). Evaluation of serum CA-125 levels in the monitoring of ovarian cancer. *Am. J. Obstet. Gynecol.*, **157**, 88.

VOEST, E.E., VAN HOWELINGEN, J.C. & NEIJT, J.P. (1989). A meta-analysis of prognostic factors in advanced ovarian cancer with median survival and overall survival (measured with the log (relative risk)) as main objectives. *Eur. J. Cancer Clin. Oncol.*, **25**, 711.

WILTSHAW, E. (1985). Ovarian cancer trials at the Royal Marsden. *Cancer Treat. Rev.*, **12** (Suppl. A), 67.